# A Compact Linear Ultrasonic Motor Composed by Double Flexural Vibrator

**DOI:** 10.3390/mi12080958

**Published:** 2021-08-13

**Authors:** Jiayin Li, Yin Wang, Ziyan Chen, Fang Cheng, Qing Yu

**Affiliations:** College of Mechanical Engineering and Automation, Huaqiao University, Xiamen 361021, China; ljy18950188761@163.com (J.L.); c_zy100@163.com (Z.C.); chf19chf19@hotmail.com (F.C.); yuqing@hqu.edu.cn (Q.Y.)

**Keywords:** linear ultrasonic motor, minimization, discrete structure optimization, performance evaluation

## Abstract

A minimized linear ultrasonic motor was proposed, and two flexural bimorph vibrators were utilized to form its stator. The construction of the linear ultrasonic motor and its operation principle was introduced. Two working modes with the same local deformation distribution were chosen on the basis of Finite Element Analysis (FEA). To obtain its optimized structural parameters, sensitivities on frequency difference were calculated, and a way of decreasing the frequency difference of two working modes was introduced. A prototype of the optimized model was made. The modal testing of the stator and its performance evaluation was conducted. The modal testing results were in good agreement with that of the simulation. The maximum speed of the prototype is 245 mm/s, and its maximum thrust is 1.6 N.

## 1. Introduction

A linear ultrasonic motor (LUSM) is an electromechanical device that transfers ultrasonic vibration with amplitude of the order of micrometers into unidirectional motion through friction coupling [1,2,3]. Since the ultrasonic vibration of LUSMs are excited by the inverse piezoelectric effect of piezoelectric ceramics, they feature fast response, high resolution, long stroke, and compact size, which make them competitors for electromagnetic counterparts [4,5,6]. 

There are several factors to classify LUSMs, including the shape of the vibrator, assembly of piezoelectric elements, and vibration modes utilized. Regarding the shape of the vibrator, there is the bar shape, plane shape, and block shape [7,8,9]. Among these shapes, the vibrators with plane shape interest researchers due to their compact size and higher efficiency [10]. In the category of plane-shaped vibrators, LUSMs can be divided into two groups regarding the relation between vibration direction and structural plane, that is, out-of-plane mode LUSMs and in-plane mode LUSMs. As the direction of the vibration displacement is perpendicular to the structural plane, it is called an out-of-plane mode LUSM, while the vibration displacement is in the structural plane, and thus, it is called an in-plane mode LUSM [11,12]. 

In recent years, LUSMs of in-plane mode have been reported. These motors are usually small and efficient [13,14]. For example, some motors that use in-plane modes as their operating modes have an output speed and weight ratio of more than 20 times (the larger the ratio, the smaller the motor, the higher the output speed) [15,16,17]. These motors all have a common feature: their operating modes are composed of two or more different types of vibration modes [18,19]. These characteristics are beneficial to single-phase excitation of the motor [20,21].

Although these merits of in-plane LUSMs are attractive for researchers, there are some challenges for designing in-plane vibrators for such LUSMs. In the existing in-plane mode LUSMs, the operating modes of the stator are different. The difference between working modes varies with the boundary conditions especially when the different vibration modes are excited, for the trend of variation may be opposite in two different vibration modes, resulting in a larger frequency gap between them [22]. Allowing for the consistency in exciting two working modes, it is beneficial for choosing working modes with the same deformation modes, which lead to the same variation when boundary conditions are changed [23,24,25].

To propose an in-plane vibrator with the same deformation mode, a minimized linear ultrasonic motor utilizing two identical local flexural vibration modes was proposed in this paper. In Section 2, the working principle of the motor was introduced. In Section 3, the motor operation mechanism was analyzed and simulated by the finite element method. The sensitivity of the motor was analyzed, and the optimization scheme was developed to reduce the frequency difference between the identical local flexural vibration modes. In Section 4, a prototype of the motor was made, and its performance was tested.

## 2. Working Principle

### 2.1. Construction

**Figure 1:**
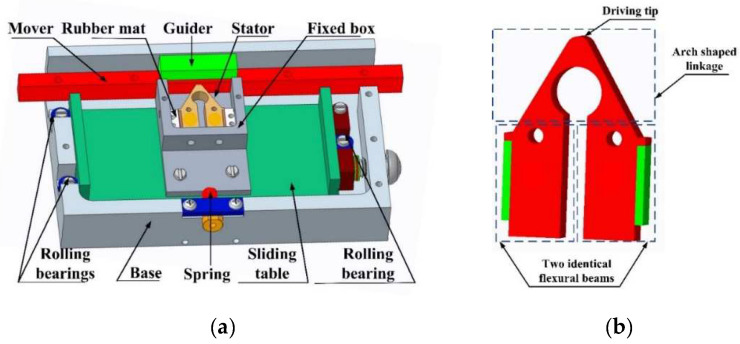
(**a**) Construction of the motor; (**b**) Stator structure.

The overall component and structure of the LUSM is shown in Figure 1a. The mechanism comprises three essential parts: a stator, a mover, and a base to which the stator and mover are fixed. The motor transfers the ultrasonic vibration of the stator into the unidirectional movement of the mover via frictional coupling. The guider, which is utilized in conjunction with the mover, balances the stator’s normal contact toward the mover and simultaneously maintains the linear shear contact. 

The frictional coupling between the stator and mover is created based on two factors:

- **Constant pre-load force being applied to normal contact**.
  Solution: The pre-loaded spring is employed to generate the pre-load force onto the stator.
- **The appliance holding the stator is able to balance the shear force between the stator and mover**.
  Solution: The roller bearing device on both sides of the base is installed to balance the force between the stator and mover.

After the above criteria have been met, the stator is stabilized inside the fixed box and clamped with a rubber mat on both sides. To achieve the force balance of the stator, a sliding table is also adopted to reinforce the fixed box. When the device is powered, the friction in between the driving tip and the mover triggers the mover to slide along the guider, consequently outputting linear movement.

Based on the stator’s function in the above-mentioned LUSM, the methodology of generating the elliptical movement of the driving tip becomes one of the main issues to be resolved during the design of the stator. As shown in Figure 1b, the structure of the stator in this thesis is composed by an arch-shaped linkage and two identical flexural beams, with two additional rectangular piezoelectric ceramic sheets attached to the outer side of both beams symmetrically to stimulate the resonance. 

### 2.2. Working Principle

**Figure 2:**
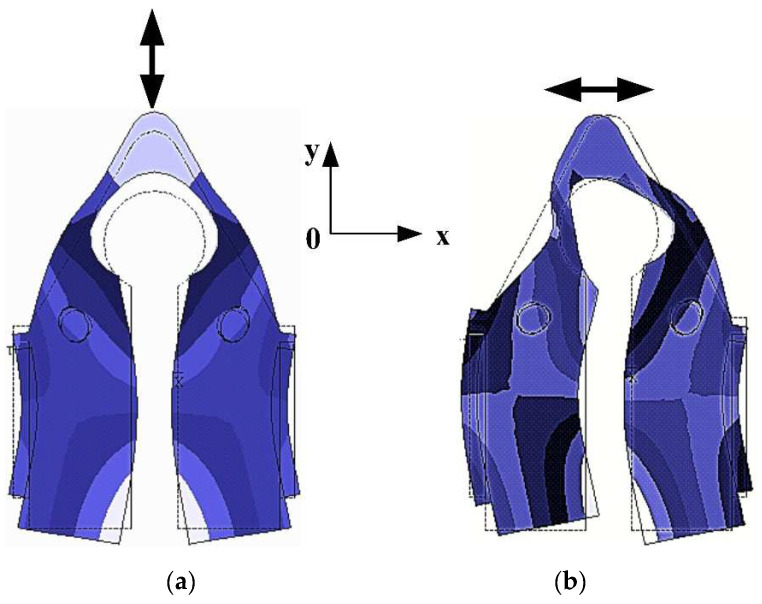
Flexural mode of the stator with patch structure: (**a**) Symmetrical flexural mode; (**b**) Anti-symmetric flexural mode.

The working mode of the stator is composed by two identical local flexural modes. When the two flexural beams vibrate in the reverse phases, the driving tip deforms and fluctuates along the *y*-axis. This is called the Symmetrical Flexural Mode, as illustrated in Figure 2a. Contrarily, when the flexural beams vibrate in the identical phase, the deformation of the beam triggers the driving tip to vibrate along the *x*-axis, which is called the Anti-symmetric Flexural Mode, as demonstrated in Figure 2b.

As shown in Figure 2, the piezoelectric ceramic sheets are pasted to the section that encounters the most strain. This configuration increases the processing efficiency upon the shearing strength in between the piezoelectric ceramic sheet and the beam structure. Under these two vibration modes in Figure 2, the driving tip generates horizontal and vertical displacement, respectively. 

The combination of these two vibration modes forms the working mode of the stator. If the two longitudinal vibration modes are under the same frequency with a particular time phase difference, the desired elliptical trajectory can be achieved via the driving tip and mover.

**Figure 3:**
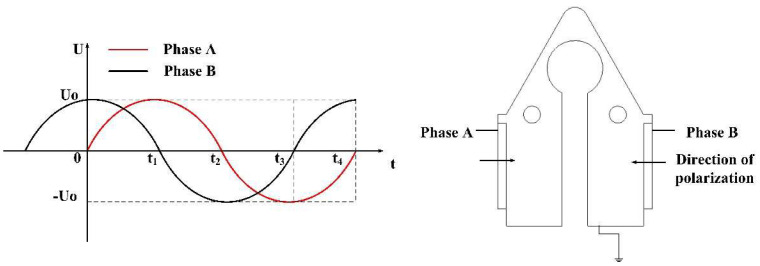
Polarization and excitation signals of the stator.

In order to trigger the bending vibration modes of these two flexural beams, the frequency range of two different voltage excitation signals were set in proximity to the frequency of the first-order bending vibration modes of the stator. These two piezoelectric ceramic sheets are both polarized along the direction of its thickness, as shown in Figure 3. A phase A sine signal is applied to the piezoelectric sheet on one side, and a phase B cosine signal, which has a 90° phase difference to phase A, has been applied to the ceramic sheet on the other side. These two signals comprise identical frequency and amplitude. The metal substrate is connecting to the ground, which indicates zero voltage potential. 

When the ceramic plate on one side of the stator is actuated by a sinusoidal voltage of phase A, the displacement response of the driving tip can be described as:

(1) $$u_{a}\left(x,\;t\right){=W}_{x}\sin\left(2\pi ft\right){+W}_{y}\sin\left(2\pi ft+\varphi \right),$$

where $W_{x}$ is the displacement response amplitude along the *x*-direction, $W_{y}$ is the displacement response amplitude along the *y*-direction, f is the excitation frequency, and *φ* is the displacement response phase shift between two working modes.

When the ceramic plate on one side of the stator is actuated by a cosine voltage of phase A, the displacement response can be expressed as:

(2) $$u_{b}\left(x,\;t\right){=W}_{x}\cos\left(2\pi ft\right){+W}_{y}\cos\left(2\pi ft+\varphi \right).$$

When dual-phase voltage signals are actuated simultaneously, the vibration trajectory of the driving tip should be the superposition of the above two vibrations, and the displacement can be derived as follows:

(3) $${\left(\frac{x}{W_{x}}\right)}^{2}+{\left(\frac{y}{W_{y}}\right)}^{2}=2\left[\sin\left(2\omega t\right)+\sin\left(2\omega t+2\varphi \right)\right].$$

When two piezoelectric ceramics are applied with two sinuous voltage signals with phase difference $\frac{\pi }{2}$, the driving tip will vibrate elliptically. When $\varphi =\frac{\pi }{2}$, the ellipse trajectory of the driving foot is Equation (4):

(4) $${\left(\frac{x}{W_{x}}\right)}^{2}+{\left(\frac{y}{W_{y}}\right)}^{2}=2.$$

**Figure 4:**
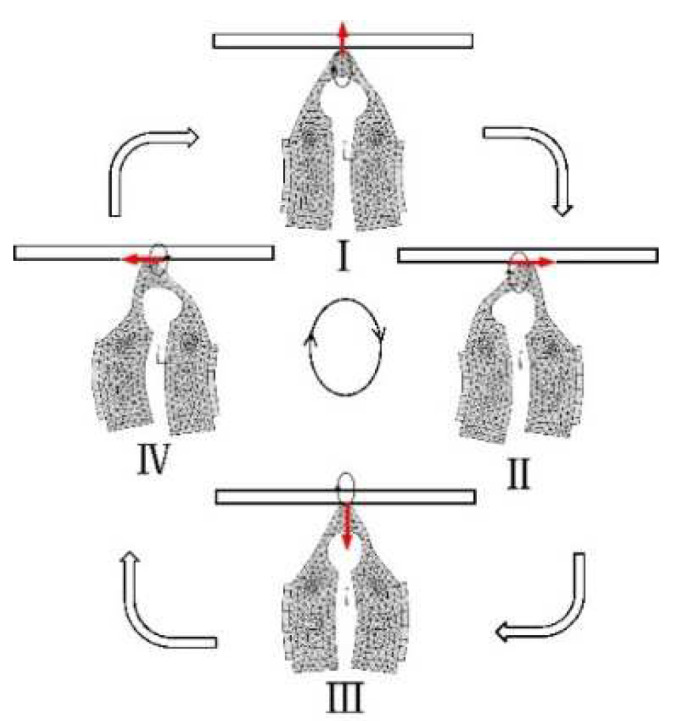
Stator actuation cycle.

The elliptical working trajectory of the beam is shown in Figure 4. The symmetrical and anti-symmetrical modes emerge alternatively, and consequently, they drive the mover via friction. When the stator vibrates in the sequence from I to IV as shown below, the driving tip rotates elliptically in the clockwise direction. The red arrow represents the direction of the driving tip.

## 3. Finite Element Analysis of Stator

In order to validate the working mode of the stator’s structure as mentioned in Section 2 and stimulate the two bending vibration modes under the same resonance frequency, the frequency difference between these two modes is required to be minimized. 

Then, the finite element method was introduced and utilized to establish the stator model by using the ANSYS software. This methodology has analyzed the impact of the stator’s size on the frequency of the bending vibration mode as well as optimized the structure of the stator. 

### 3.1. Modeling and Simulation

**Figure 5:**
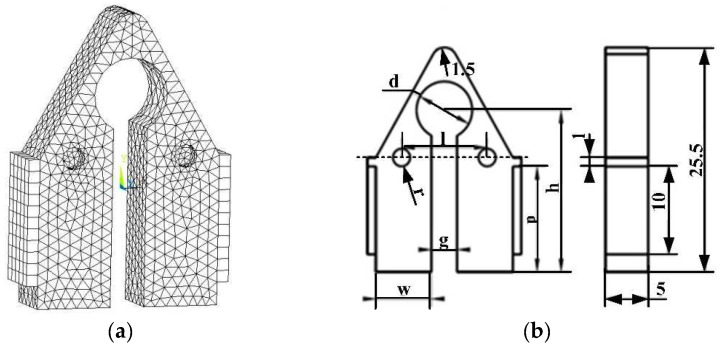
The structure parameters and finite element model of the excitation-coupled stator of the patch structure: (**a**) Finite element model; (**b**) The parameters of the structure.

The finite element model as detailed in Section 2 is demonstrated in Figure 5a. The 22,802 nodes on the stator model were selected to extract the modal shapes. A Solid5 element was used for meshing the piezoelectric ceramics and a Solid45 element was applied for meshing the metallic matrix. The number of tetrahedral elements was 7788. The mechanical boundary condition was set as free, and the potential of the piezoelectric ceramic substrate was determined as zero. The specific simulation time is 6 s. The initial geometrical dimensions of the stator’s structure are illustrated in Figure 5b and Table 1. 

**Figure 6:**
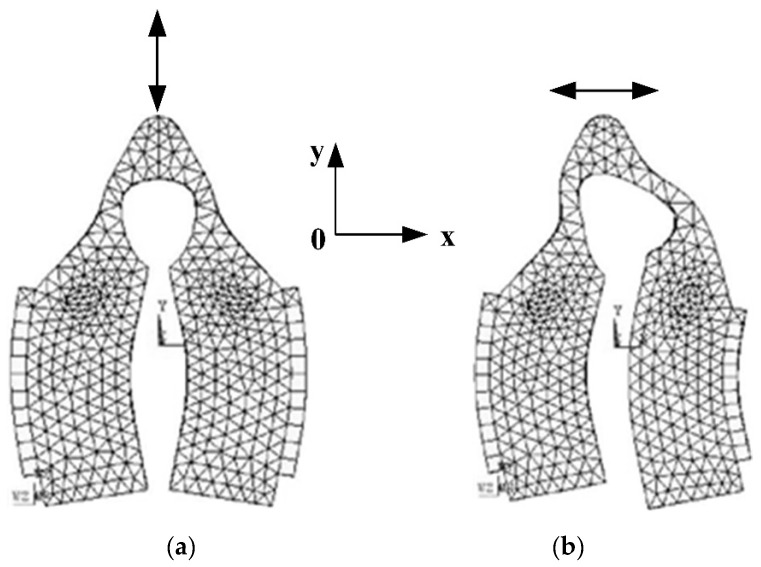
The stator finite element model of the patch structure: (**a**) Symmetrical mode frequency 71,454 Hz; (**b**) Anti-symmetrical modal frequency 70,334 Hz.

The outcome from the ANSYS simulation has further proved the working modes of the stator described in Section 2. By adjusting the structural parameter, the resonance frequencies for these two working modes were 71,454 Hz and 70,334 Hz (1120 Hz in difference), as shown in Figure 6. The formation of the elliptical working trajectory relies on the minimization of the frequency difference between the two working modes. 

Since the frequency difference of the symmetrical and anti-symmetrical modes is still considered substantial, the structural parameter of the stator is required to be further modified and optimized.

### 3.2. Structure Optimization

The sensitivity analysis was employed to reduce the frequency difference between the two working modes by altering the structural parameters of the stator.

To minimise the frequency difference within the two working modes mentioned in Section 3.1, the sensitivity analysis was adopted upon the parameters that have greater impacts on the working mode of stator. The corresponding expression for sensitivity can be written as: [26]:

(5) $$S_{(a,b)j}=\frac{\frac{\partial f_{(a,b)j}}{P_{j}}}{\frac{\partial P_{j}}{P_{j}}}=\frac{\Delta f_{(a,b)j}}{\Delta P_{j}},$$

where $f_{aj}$ is the frequency of the symmetric mode and $f_{bj}$ is the frequency of the anti-symmetric mode. $P_{j}$ represents the structural parameters. $\Delta f_{aj}$ is the small change of symmetric working mode frequency and $\Delta f_{bj}$ is the small change of anti-symmetric working mode frequency, where $\Delta P_{j}$ is the small change of the stator structure $P_{j}$. The sensitivity of modal frequency difference to the structural parameters is:

(6) $$S_{fj}=S_{(a,b)j}=\frac{\partial f_{(a,b)j}}{\partial P_{j}}=\frac{\left(f_{aj}-f_{bj}\right)-\left(f_{aj0}-f_{bj0}\right)}{\Delta P_{j}},$$

where $\;f_{aj}{,\;f}_{bj}$ are modal frequencies of symmetrical modes and anti-symmetrical mode before changes of geometrical parameters $P_{j}$. $f_{aj}{,\;f}_{bj}$ are the modal frequencies of the symmetrical modes and anti-symmetrical mode after changes of geometrical parameters $P_{j}$. Using the finite element model in Section 3.1 and Equation (6) to calculate the geometric parameters of the stator, the sensitivity of frequency difference to each geometric parameter can be obtained.

**Figure 7:**
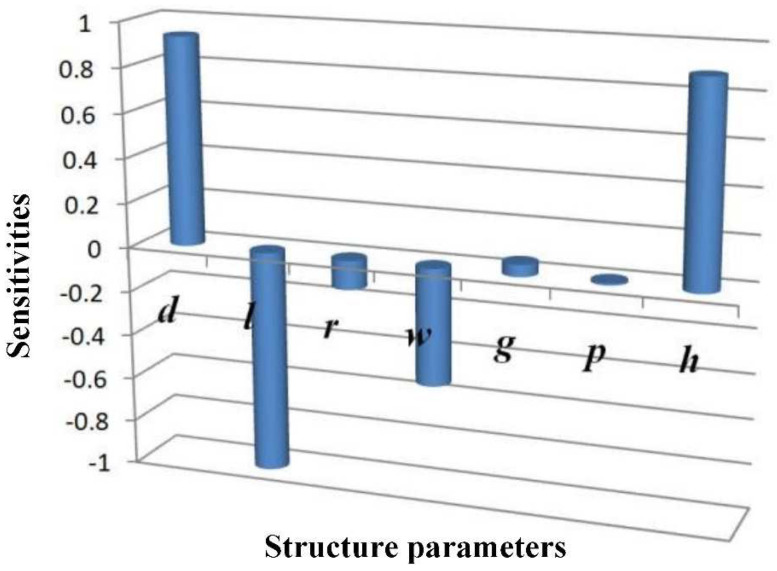
Structural parameter sensitivity.

The result from the sensitivity analysis is shown in Figure 7. In comparison, structural parameters ***d***, ***l***, ***w***, and ***h*** have a more substantial influence than other parameters on the working modes. By adjusting these parameters and conducting another simulation, the frequency difference was proven been further reduced to a minimum of 840 Hz.

The frequency difference of 840 Hz is not conducive to the stability of the motor; hence, apart from only adjusting the structural parameters, the design concept and methodology of the stator optimization are also required to be further assessed. In accordance with the sensitivity analysis, the central distance in between the symmetrical opening also impacts on the working mode frequency. 

**Figure 8:**
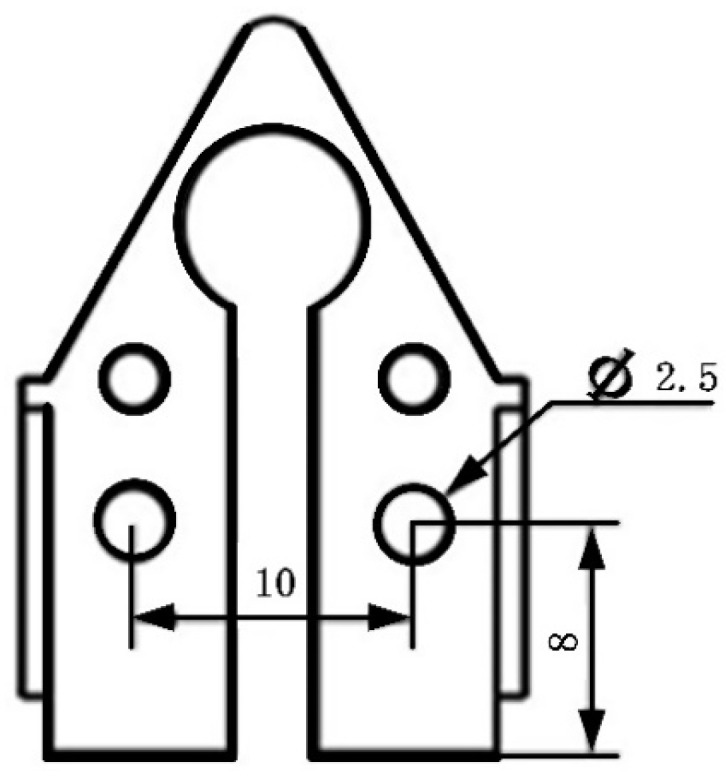
Open hole to optimize stator dynamic characteristics.

By introducing new openings on the stator (as shown in Figure 8) and implementing another simulation, the frequency difference was further reduced to less than 840 Hz. 

**Figure 9:**
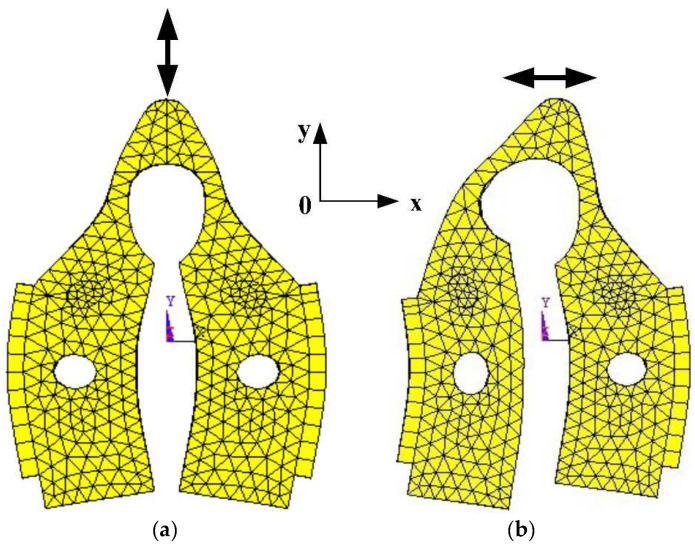
Modal analysis after optimized structure: (**a**) Anti-symmetrical modal analysis; (**b**) Symmetrical modal analysis.

**Figure 10:**
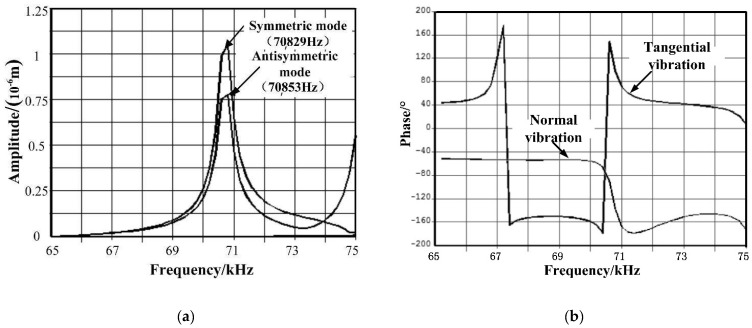
Harmonic response analysis of stator: (**a**) Amplitude frequency response; (**b**) Phase frequency response.

Then, the finite element model was reviewed and re-established based on the modified parameters, and further modal analysis was developed upon the optimized stator. Figure 9 demonstrates the final structural dynamic. The frequency of the symmetrical mode is 70,829 Hz, and for the anti-symmetrical mode, the frequency resulted in 70,853 Hz, which only has 24 Hz in difference. It ensured the effectiveness and validity of the analysis on the working mode. The amplitude and phase frequency response collected from the surface nodes on the driving feet are illustrated in Figure 10.

**Figure 11:**
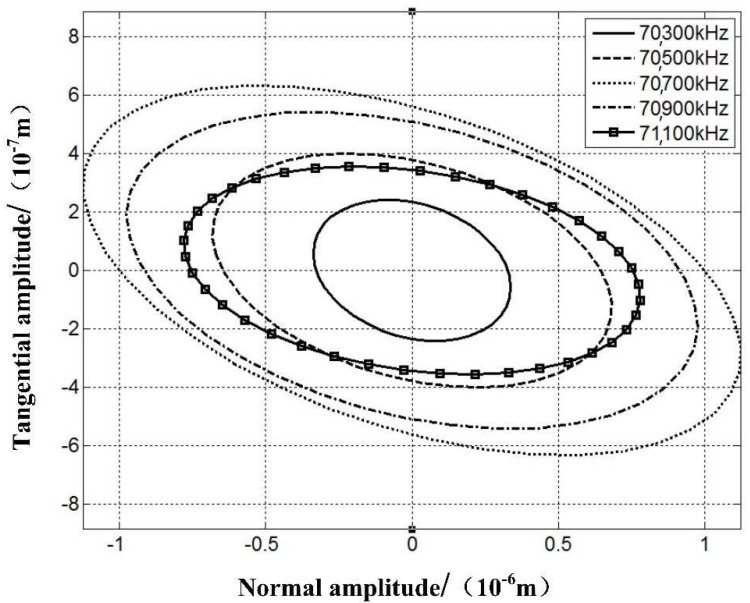
Feet trajectory driven by harmonic response.

To verify the effectiveness of the optimized stator, two voltage signals, phase A and phase B, have been applied to the piezoelectric ceramic sheets, and harmonic analysis was conducted in accordance with the finite element model. The phase difference between these two voltage signals is $\frac{\pi }{2}$, and the amplitude is 150 V, as shown in Figure 11.

The elliptical trajectory, in terms of its phase shift and amplitude, deforms in accordance with the frequency of the voltage signal. The trajectory is derived from the vibration response of the superposition of the two working modes. When different voltage signals are implemented with separate frequencies, the vibration response of the superposition also changes, and it consequently generates various elliptical trajectories with different sizes and directions. As the ratio between the phase shifts of these two modal frequency responses approaches 1, the ellipse becomes flatter. 

The result indicates that under the voltage simulation with a 90° phase difference, the working track of the driving tip is not an absolute ellipse but rather shifting along the direction of half axis based on the frequency.

The optimized stator has now achieved the desired elliptical trajectory. By considering and analyzing the amplitude and frequency of the stator, as well as the phase frequency response characteristic, the stimulation frequency of the voltage signal has a significant impact on the driving tip’s working track, and it consequently affects the output of the motor. In the practical working environment, due to the rise of temperature, drifting of the driver frequency, and other potential factors, the vibration of the stator will be consistently altered and hence lose the stability and controllability of the motor. 

## 4. Experimental Study

### 4.1. Vibration Characteristic Experiments

To validate the function of the optimized stator in Section 3, the vibration mode of the stator and the output characteristic of the motor were both experimented separately to conduct observation and assess the workability of the stator. If the stator’s vibration mode is identical to the simulation, it will consequently verify the vibration characteristic of the stator as well as the validity of the design.

The experiment was established by utilizing the Renishaw XL-80 (Renishaw, Wotton-under-Edge, UK) laser interferometer.

By observing the stator simulation as mentioned in Section 3, the lateral side of the stator encounters the most deformation of bending. In addition, due to the symmetrical feature of the stator, the vibration at the underside of the two flexural beams has sufficiently indicated the two working modes (i.e., Symmetrical and Anti-Symmetric Flexural Mode). Thus, the lateral and bottom side of the stator were selected to conduct the vibration characteristic experiment. The experimental procedure is below:

- Set the scanning point on the sample;
- Establish grids on the testing surface of the stator;
- Apply the voltage signal as described in Section 2.2 and assess the motor’s working mode.

The color change and amplitude variation of the grid reveals the vibration status of the testing face. When the testing face achieves the most bending deformation, the color of the corresponding grid changes to red, which indicates the peak value of the bending mode. Contrarily, the grid shown in blue demonstrates the nadir of the modal. 

**Figure 12:**
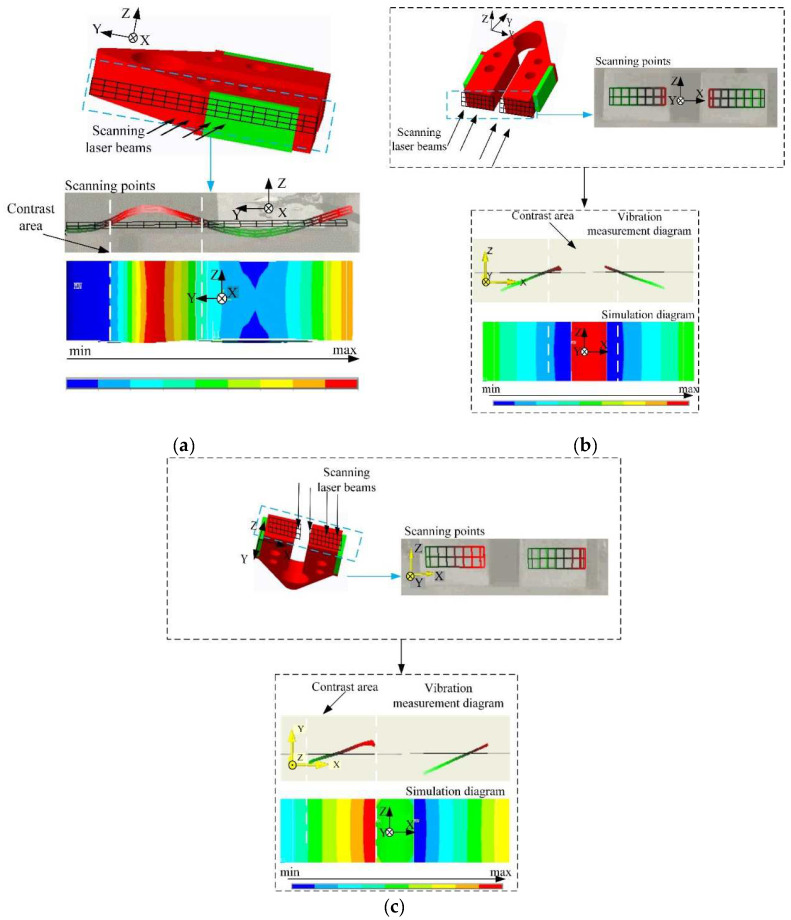
(**a**) Comparison of the test vibration cloud image and simulation mode on the stator side; (**b**) Comparison of vibration cloud image and simulation mode of the symmetrical mode test on the underside of the stator; (**c**) Comparison of vibration cloud image and simulation mode of an antisymmetric modal test on the underside of the stator.

As shown in Figure 12a, the modal on the lateral side resulted in a sine wave, which is identical to the first-order bending mode in the simulation. Hence, the vibration mode of the stator was testified to be the local bending mode. Figure 12b,c illustrates the vibration at the underside of the stator, which has reached the peak value at the center and both ends, which jibes to the Symmetrical and Anti-Symmetrical Flexural Mode as shown in Figure 9, given that the vibration amplitude changes uniformly. 

The above experimental outcome has verified the validity of the design as well as the vibration characteristic of the stator. Furthermore, the frequency gap of the working mode and the difference in the theoretical value also form part of the assessment criteria for the stator’s functionality. 

**Figure 13:**
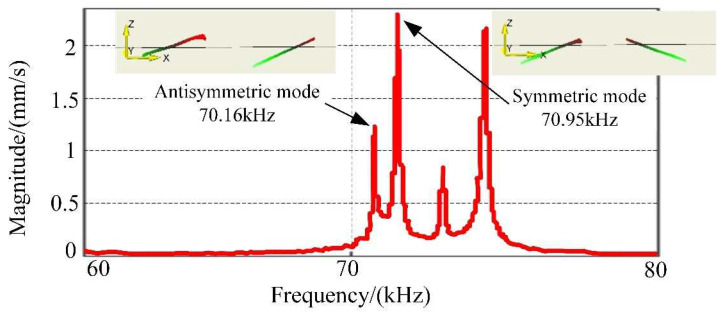
The frequency response curve of the stator.

Figure 13 shows the amplitude-frequency chart established based on the experimental data. The motor frequencies of the two working modes are 70.95 Hz and 70.16 Hz. Comparing to the finite element model in Figure 9 (70.829 Hz and 70.853 Hz), the relative error is 0.17% for Symmetrical Mode and −0.97% for Anti-Symmetrical Mode. The potential root causes are listed as below:

- The rubber packer was utilized to stabilize the stator, which in fact simplified the device;
- The boundary condition is not identical for practical experiments and lab simulation;
- Geometrical tolerance during the manufacturing process for the prototype motor can also contribute to this error;
- The material of the prototype can potentially vary from the design in terms of the uniformity and technical parameter.

### 4.2. Performance Evaluation

**Figure 14:**
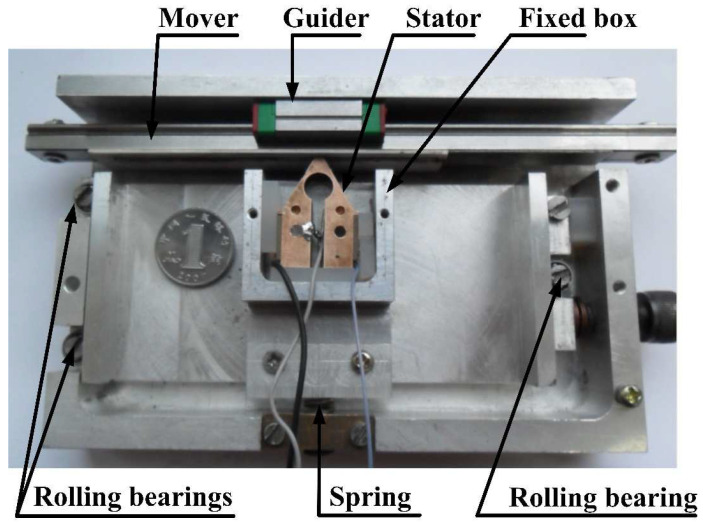
The actual motor.

The experimental error of the working mode frequency in Section 4.1 is within the tolerance, hence indicating that the design of the stator conforms to the design criteria and performs steadily. Based on the result of the finite element simulation, a prototype was fabricated to verify the functionality. The actual motor being tested during the experiment was illustrated in Figure 14. An optimized stator was placed in the fixed box, and all other components are same as the structure figure in Section 2.

**Figure 15:**
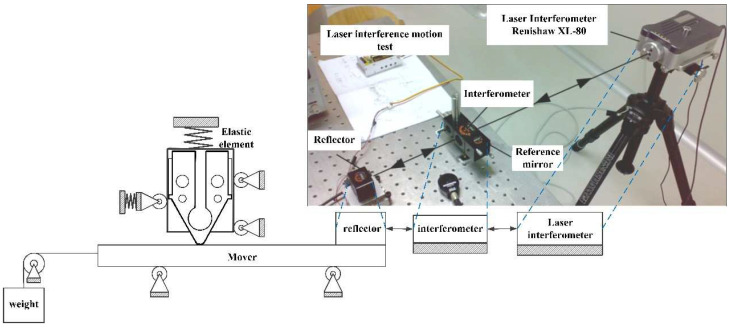
Output characteristic test system and laser interference motion test system.

The overall setup of the experiment was illustrated in Figure 15. The Renishaw XL-80 (Renishaw, Wotton-under-Edge, UK) laser interferometer was applied to measure the vibration of the stator. A heavy mass object was suspended from the mover to simulate the load via rope and pulley.

**Figure 16:**
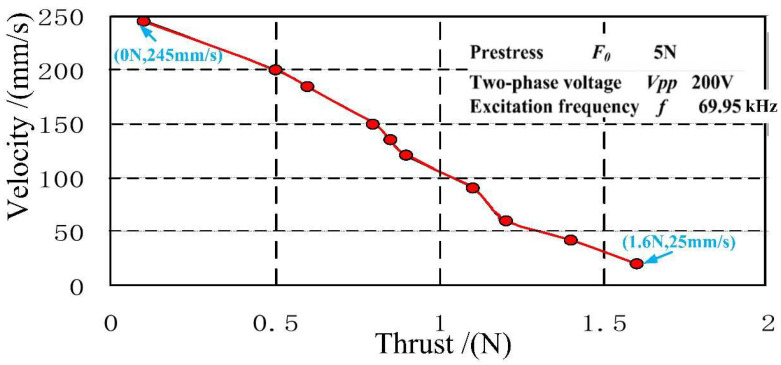
The influence of load on the motor output speed.

The experiment was conducted inside a Class 100,000 cleanroom. The reflector moves simultaneously with the mover. The mechanical performance curve is shown in Figure 16. The data were captured when the voltage signal frequency reached 69.95 kHz. 

In accordance with the mechanical performance curve, the output voltage from the motor was evaluated to be 300 Vp-p under 5 N of load. The maximum speed reached 245 mm/s when no load was applied. The minimum speed in the test reached 25 mm/s when 1.6 N load was applied.

This result differed from the simulation analysis, which can be interpreted from the perspective of boundary conditions. The driving tip was under stress from the spring, and a silicone pad was applied to both the lateral and bottom side of the stator to distribute the stress. These factors have resulted in the experimental error comparing to the simulation result.

The friction between the driving tip and mover has impacted on the output efficiency of the motor. A potential solution is to add the friction pair to increase the friction resistance, hence enhancing the wear resistance of the driving tip. 

Since the main objective of this thesis is to testify the working principal, hence, the driving tip was not treated regarding the wear-and-tear. To optimize the contacting face between the driving tip and mover, the aluminum oxide ceramic coating will be applied to the surface of the mover.

## 5. Conclusions

In this paper, a criterion is proposed in which working modes with the same deformation mode can reduce the frequency gap between vibration modes. According to the criterion, a minimized linear ultrasonic motor utilizing two identical local flexural vibration modes was proposed. The working principle of the motor was analyzed, and the finite element method was used to simulate it. The stator structure was optimized according to the sensitivity analysis of various parameters. The relationship between the load and speed of the optimized motor was tested. The weight of the miniature piezoelectric motor is about 3 g. The resonance frequency of the motor has been designed and tested to be approximately 70.8 kHz. The maximum speed of the prototype is 245 mm/s, and its maximum thrust is 1.6 N. Since the present prototype was designed without considering the wear of the driving tip and the influence of the clamping mechanism on the consistency between working modes, the following research will focus on improving the life span and a better clamping mechanism.

## Figures and Tables

**Table 1:** Dimensions of the stator (unit: mm).

Parameters	*d*	*l*	*r*	*w*	*g*	*p*	*h*
**Values**	6.5	10.0	1.0	6.5	3.0	12.0	18.38
